# Memory Café in Rural Communities: Reflection from Participatory Action Research (PAR)

**DOI:** 10.1002/alz.088598

**Published:** 2025-01-09

**Authors:** Eric Li

**Affiliations:** ^1^ University of British Columbia ‐ Okanagan campus, Kelowna, BC Canada

## Abstract

**Background:**

Memory Café, introduced by Dr. Bere Miesen in 1997, has become a global initiative fostering connections among individuals affected by dementia. This community‐based platform facilitates interactions between dementia patients, caregivers, professionals, and the wider public, allowing for open sharing of experiences. Despite its global success, there remains a gap in research regarding its implementation in rural and remote regions.

**Method:**

Our participatory action research aimed to investigate the impact of Memory Café in interior British Columbia, Canada, over a six‐month period. A multi‐disciplinary team collaborated with stakeholders in three rural communities, employing a multi‐method design to explore various experiences and insights. The research identified three key themes.

**Results:**

Firstly, the study highlighted the importance of “listening” and the creation of “inclusive spaces” in rural and remote communities. Due to heightened social isolation, patients and caregivers expressed a need for spaces like Memory Café, enabling them to openly share challenges. Emphasizing the significance of listening in Indigenous settings, this theme underscored the health benefits for both individuals and communities.

Secondly, operational challenges faced by initiatives like Memory Café were revealed. Uncertainties related to financial and human resources, including volunteers, significantly impacted sustainability in resource‐limited areas. Gaps in government programs and policies were identified, emphasizing a lack of comprehensive support for patients and caregivers.

Lastly, the research highlighted the role of creativity and intergenerational outreach. Arts‐based methodologies were well‐received by participants, presenting an opportunity for innovation.

**Conclusion:**

In conclusion, our research sheds light on both the benefits and challenges of Memory Café in rural and remote communities. Future studies should prioritize exploring mechanisms to sustain community‐based initiatives and develop holistic support systems for dementia patients and caregivers in non‐urban settings.

